# Modeling of nitrogen fixation and polymer production in the heterotrophic diazotroph *Azotobacter vinelandii* DJ

**DOI:** 10.1016/j.mec.2020.e00132

**Published:** 2020-05-30

**Authors:** Diego Tec Campos, Cristal Zuñiga, Anurag Passi, John Del Toro, Juan D. Tibocha-Bonilla, Alejandro Zepeda, Michael J. Betenbaugh, Karsten Zengler

**Affiliations:** a Department of Pediatrics, University of California, San Diego, 9500 Gilman Drive, La Jolla, CA, 92093-0760, USA; b Facultad de Ingeniería Química, Universidad Autónoma de Yucatán, Mérida, Yucatán, Mexico; c Department of Chemical and Biomolecular Engineering, The Johns Hopkins University, 3400 North Charles Street, Baltimore, MD, 21218, USA; d Bioinformatics and Systems Biology Graduate Program, University of California, San Diego, La Jolla, CA, 92093-0412, USA; e Department of Bioengineering, University of California, San Diego, La Jolla, CA, 92093-0412, USA; f Center for Microbiome Innovation, University of California, San Diego, 9500 Gilman Drive, La Jolla, CA, 92093-0403, USA

## Abstract

Nitrogen fixation is an important metabolic process carried out by microorganisms, which converts molecular nitrogen into inorganic nitrogenous compounds such as ammonia (NH_3_). These nitrogenous compounds are crucial for biogeochemical cycles and for the synthesis of essential biomolecules, i.e. nucleic acids, amino acids and proteins. *Azotobacter vinelandii* is a bacterial non-photosynthetic model organism to study aerobic nitrogen fixation (diazotrophy) and hydrogen production. Moreover, the diazotroph can produce biopolymers like alginate and polyhydroxybutyrate (PHB) that have important industrial applications. However, many metabolic processes such as partitioning of carbon and nitrogen metabolism in *A. vinelandii* remain unknown to date.

Genome-scale metabolic models (M-models) represent reliable tools to unravel and optimize metabolic functions at genome-scale. M-models are mathematical representations that contain information about genes, reactions, metabolites and their associations. M-models can simulate optimal reaction fluxes under a wide variety of conditions using experimentally determined constraints. Here we report on the development of a M-model of the wild type bacterium *A. vinelandii* DJ (*i*DT1278) which consists of 2,003 metabolites, 2,469 reactions, and 1,278 genes. We validated the model using high-throughput phenotypic and physiological data, testing 180 carbon sources and 95 nitrogen sources. *i*DT1278 was able to achieve an accuracy of 89% and 91% for growth with carbon sources and nitrogen source, respectively. This comprehensive M-model will help to comprehend metabolic processes associated with nitrogen fixation, ammonium assimilation, and production of organic nitrogen in an environmentally important microorganism.

## Highlights

• Genome-scale metabolic model of *Azotobacter vinelandii* DJ achives over 90% accuracy.
• *i*DT1278 is the most comprehensive model to simulate diazotrophy.
• Determining the most suitable culture conditions to produce polymers *A. vinelandii*.
• Constraint-based modeling unravels links among nitrogen fixation and production of organic nitrogen.

## 1 Introduction

*Azotobacter vinelandii* is a gram-negative soil bacterium capable of converting atmospheric nitrogen gas (N_2_) into soluble ammonia (NH_3_) as well as into other important nitrogenous compounds (Gyurján et al., 1995; Howard and Rees, 1996). *Azotobacter* and related *Azospirillium* are estimated to fix up to 10–30% of the total nitrogen in the rhizosphere (Cleveland et al., 1999). Nitrogen fixation can be carried out under ambient conditions by any of the three highly specialized metal-dependent nitrogenases, referred to as molybdenum nitrogenase, vanadium nitrogenase, and iron-only nitrogenase (Setubal and Almeida, 2015; Sippel et al., 2017). Nitrogenases produce high concentrations of fixed ammonium, which is excreted and serves as essential nutrient for other organisms (Ambrosio et al., 2017; Tan et al., 2015). However, the activity of these enzymes is highly sensitive to molecular oxygen and energetically costly. Diazotrophs, such as *A. vinelandii* have developed specific strategies to protect the nitrogenase complex in diazotrophic conditions (Setubal and Almeida, 2015). One of the most studied mechanisms for nitrogenase protection is alginate biosynthesis. Alginate is transported to the extracellular space where it works as a barrier that decreases oxygen diffusion into the cytoplasm and thus maintains high functionality of oxygen-sensitive nitrogenases in anoxic environments (García et al., 2018; Pacheco-Leyva et al., 2016).

Alginate is of great industrial value because of its use as biocompatible and biodegradable exopolysaccharide. This polymer is used as gel-film-stabilizing, -thickening, or -forming agent in the food and pharmaceutical industry (Remminghorst and Rehm, 2006). Besides alginate bioproduction, *A. vinelandii* produces another attractive commercial polymer, i.e. polyhydroxybutyrate (PHB) (Yoneyama et al., 2015). PHB is synthetized by this microorganism under high carbon/nitrogen ratios as a carbon and energy reserve in the form of cysts (Stevenson and Socolofsky, 1966; Zúñiga et al., 2011). Both biopolymers can be produced in elevated concentrations, representing 30–70% of the dry biomass (Pacheco-Leyva et al., 2016; Yoneyama et al., 2015).

*A. vinelandii* has been shown to grow under a broad range of heterotrophic conditions and is able to metabolize, different sugars, alcohols, and organic acids as well as nitrogen-containing compounds (Nagai et al., 1972; Quiroz-Rocha et al., 2017; Sahoo et al., 2014; Shawky et al., 1987). Despite this metabolic versatility to use different carbon and nitrogen sources, several of the internal metabolic processes regarding carbon and nitrogen partitioning (division and distribution of an element into metabolic, structural or storage pools) in *A. vinelandii* remain unknown. Today there are five fully sequenced genomes available for *A. vinelandii* strains (e.g. *A. vinelandii* CA, DJ, CA6, DSM 279, and NBRC13581) (Noar et al., 2015; Setubal et al., 2009; Setubal and Almeida, 2015), enabling a comprehensive functional characterization of *Azotobacter* metabolism at genome-scale.

To comprehend the metabolic capabilities of *Azotobacter vinelandii* DJ we used a systems biology approach, which offers tools to predict the organism behavior based on mathematical representations of biological data. M-models can be reconstructed using semi-automated tools that generate a draft model. This draft model is further curated manually to increase its quality. To date, only two core M-models of *Azotobacter vinelandii* are available that contain a reduced number of metabolic reactions. These core reactions are in general related to nitrogen fixation or PHB and alginate production, disregarding most of the central metabolism of the microorganism (e.g. TCA cycle, lipid metabolism and some amino acids synthesis) (García et al., 2018; Inomura et al., 2018). Here we have developed a M-model for *Azotobacter vinelandii* DJ to contextualize metabolic processes associated with nitrogen fixation, ammonium assimilation, and production of organic nitrogen on genome-scale. Our model was successfully validated using high-throughput phenotypic data and physiological data.

## 2 Material and methods

### 2.1 Draft model generation

The draft model of *A. vinelandii* DJ was generated using The COBRA (Heirendt et al., 2019) and The RAVEN (Agren et al., 2013) Toolboxes. The proteome sequence was obtained from PATRIC database (Genome ID: 322710.5) and was used as input sequence to reconstruct the draft model based on protein homology. We selected five reference models as templates after alignment of the complete genome sequences of *A. vinelandii* DJ with all bacteria with available models in the BiGG Database (King et al., 2016). Templates included *Escherichia coli* str. K-12 substr. MG1655, model *i*ML1515 (Monk et al., 2017), *Klebsiella pneumoniae* subsp. pneumoniae MGH 78578, model *i*YL1228 (Liao et al., 2011), *Geobacter metallireducens* GS-15, model *i*AF987 (Feist et al., 2014), *Clostridium ljungdahlii* DSM 13528, model *i*HN637 (Nagarajan et al., 2013), and *Methanosarcina barkeri* str. Fusaro, model *i*AF692 (Feist et al., 2006). Template models contained reactions associated with nitrogen fixation, H_2_ production, acetate consumption, amino acids catabolism and sugar degradation (Fig. 2). The generated draft model also contained genes (exogenous genes) from template models, which were later removed during the manual curation step.

### 2.2 Model refinement

#### 2.2.1 Manual curation

We used PATRIC (Wattam et al., 2017) to identify essential genes for *A. vinelandii DJ* in the final model. We only extracted those genes that had a given Enzyme Commission (EC) number that could be used to obtain the GPR (gene-protein reaction) associations. The final list of reactions with EC number and gene association not previously present in the model were balanced and added to the model before analyzing GPR associations.

Model refinement included two major steps: manual curation/review of the GPR associations and gap-filling by adding new metabolic reactions in the model. In the first step of manual curation, we determined sequence similarity among *A. vinelandii DJ* proteins and the exogenous proteins in the GPRs to identify *A. vinelandii* (AVIN) genes closely related to the exogenous proteins. We identified proteins based on BLASTp criteria of ​≥ ​40% identity, e-value ​≤ ​1e^−4^, and query coverage ​≥ ​85%. A second step of manual curation was performed based on protein function, type of metabolic reaction, and GPR associations. Then all the GPR associations were manually curated to catalyze biological reactions that they were associated with. PATRIC essential genes previously identified were added in this step of manual curation. Remaining reactions with mixed AVIN and exogenous genes in the GPR association were manually curated in order to remove the genes that did not belong to *A. vinelandii*. Reactions with exclusively exogenous GPR associations were identified through previous manual curation steps. Afterwards, Flux Balance Analysis (FBA) was performed to identify which of these reactions carry any flux under experimental conditions (Orth et al., 2010). From these evaluated reactions, those with no flux and exogenous GPR associations were removed from the model.

#### 2.2.2 Gap-filling

Gap-filling analysis was performed to identify the metabolites disconnected in the model. These metabolites were classified depending on the number of reactions present in the model or their capability to be consumed, produced, or both. Disconnected reactions were manually curated using information from different bioinformatic databases (e.g. KEGG, Biocyc). From these results, gap-filling was used to connect pathways through the data retrieved. A second step of gap-filling was accomplished to connect the metabolites from the medium conditions retrieved using literature information (Wong and Maier, 1985) through algorithms to identify the reactions involved in the carbon source assimilation. A total of 38 carbon sources were used under nitrogen fixation and ammonium assimilation conditions. Complementary, experimental data were generated using Biolog plates to test different carbon and nitrogen sources. This was employed to improve the quality of model predictions under a wide variety of conditions. A set of 190 carbon sources and 95 nitrogen sources were used to connect the networks properly. Subsequently, the GPR associations were verified for each reaction added during the gap-filling to maintain the quality of the model. Those reactions with no gene information and literature validation were conserved as orphan reactions.

#### 2.2.3 Final quality control and quality analysis

Final quality check was performed by a person who did not perform the manual curation to assess the quality of the data. We performed *in-silico* GPR simulations to verify if the GPR associations are correctly assigned using the COBRA Toolbox algorithms. Next, we performed Mass Balance simulations on the model to check for unbalanced reactions added during the model refinement. Ultimately, the final model was tested looking for ATP, NADH, and NADPH free energy production, removing exchange reactions, and calculating their accumulation.

### 2.3 Constraints and growth simulations

Experimental data from the literature were retrieved to calculate the initial medium constraints. For each growth condition, the carbon, nitrogen, and hydrogen fluxes were initially determined depending on every value obtained from the literature. The constraints related to mineral compounds and exchange reactions are summarized in Table S1. Initially, a set of six different conditions were used to measure the accuracy of the model. The carbon sources verified in this stage of validation were carbohydrates under nitrogen fixation or ammonium assimilation conditions. The simulation results were compared to this set of experimental values to identify the quality in the model predictions. Subsequently, 38 carbon sources under nitrogen fixation and H_2_ consumption conditions from the literature (Wong and Maier, 1985) were used to test and increase the quality of the model. The uptake rates were estimated from the experimental conditions and set for all the carbon sources; nitrogen and H_2_ uptake rates were not fixed to a specific value according to the experimental conditions. Finally, model benchmarking was performed for 190 carbon sources (Biolog plates PM1 and PM2) and 95 nitrogen sources (Biolog plates PM3) to validate model predictions. Biolog microplates experiments to test carbon and nitrogen assimilation were performed in the present work, measuring the growth rate values in the plate reader for 96 ​h. For carbon sources evaluation, ammonium assimilation was not fixed to a specific value (non-diazotroph conditions). The experimental results from Biolog plates were matched with data retrieved from the literature to determine and evaluate model precision during the simulations. During the nitrogen condition simulations, pyruvate was used as the unique carbon source. Statistical parameters were calculated according to the comparison between the metabolic predictions and the experimental values. The model accuracy from the Biolog plates results was compared with the *in-silico* predictions of the *A. vinelandii* model from CarveMe to identify the quality of the model simulations of the present work. The alginate production capability of the model was tested using four different carbon sources (carbohydrates) from the literature (Revin et al., 2018). The carbon compound uptake rates were calculated according to the experimental values. The simulations were performed initially setting ammonium as unique nitrogen source and subsequently molecular nitrogen was established as the unique nitrogen source. Furthermore, the predicted values were compared to determine which conditions allow a higher alginate production rate. For polyhydroxybutyrate (PHB) production, the metabolic internal fluxes for the principal pathways related to the PHB synthesis were calculated (glycolysis, pentose phosphate pathway, Entner-Doudorrff pathway, and TCA cycle) and compared with fluxomic data determined by Wu et al. (2019). *In silico* predictions were performed through FBA, using The COBRA Toolbox and the Gurobi Optimizer v.8.0.1 solver (Gurobi Optimization) for MATLAB (MathWorks). Percent error between experimental values and *in silico* results were calculated to obtain model accuracy.

### 2.4 Carbon and nitrogen partitioning analysis

Experimentally determined growth phenotypes using Biolog plates were used to validate predicted carbon and nitrogen flux distributions. The internal fluxes for all the reactions of the model were calculated *in-silico* for all the carbon sources (PM1 and PM2) experiments. The reactions were grouped in general subsystems that represented the complete metabolism of *A. vinelandii* DJ. Subsequently, an average flux per subsystem was calculated using the flux values of all the reactions belonging to the subsystem. This procedure was performed to calculate the carbon and nitrogen distribution in each general subsystem under diazotrophic and non-diazotrophic conditions. Ultimately, the carbon and nitrogen distributions (the grouped average fluxes per subsystem) were compared through a linear correlation analysis in order to determine how the fluxes change through the experimental conditions for carbon sources set in the Biolog plates. High linear correlation coefficients (r) indicate a similar distribution of a specific subsystem (above 0.9), while low coefficients suggest different carbon or nitrogen distributions when comparing diazotroph and non-diazotroph conditions.

## 3 Results

### 3.1 Metabolic network reconstruction of *A. vinelandii* DJ

**Fig. 1:**
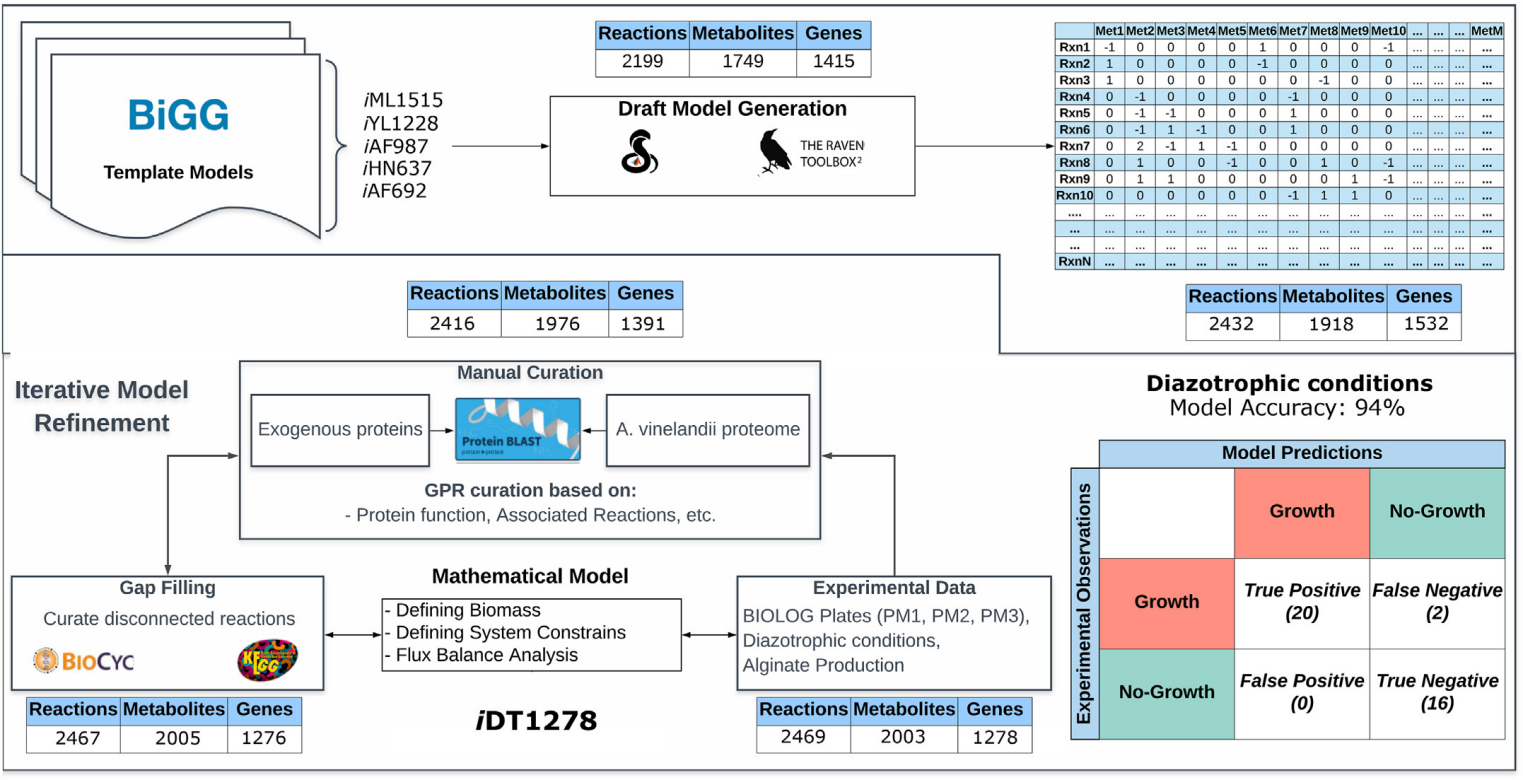
Workflow used to reconstruct a metabolic model of *A. vinelandii* DJ. A draft model was created from five template models present in BiGG (*Escherichia coli* str. K-12 substr. MG1655, *Klebsiella pneumoniae* subsp. pneumoniae MGH 78578, *Geobacter metallireducens* GS-15, *Clostridium ljungdahlii* DSM 13528 and *Methanosarcina barkeri* str. Fusaro). The RAVEN toolbox Agren et al., 2013 for MATLAB was used to create the draft model from stoichiometric data. The initial draft model contained 2,432 reactions, 1,918 metabolites, and 1,532 genes. The iterative process of model refinement included manual curation, gap-filling and curation using experimental data. The resultant final model contained *A. vinelandii* specific metabolic processes such as nitrogen fixation, and production of alginate and PHB. The final model, containing 2,469 reactions, 2,003 metabolites, and 1,278 genes, predicted with 94% accuracy.

We used a semiautomatic approach to reconstruct the M-model of *A. vinelandii* DJ (Fig. 1). This approach has been previously applied for the reconstruction of M-models (Zuñiga et al., 2016). First, a draft model of *A. vinelandii* DJ was reconstructed using the genome annotation from PATRIC (Genome ID: 322710.5). Five manually curated and validated M-Models were used as protein homology templates: *Escherichia coli* str. K-12 substr. MG1655 (Monk et al., 2017), *Klebsiella pneumoniae* subsp. pneumoniae MGH 78578 (Liao et al., 2011), *Geobacter metallireducens* GS-15 (Feist et al., 2014), *Clostridium ljungdahlii* DSM 13528 (Nagarajan et al., 2013), and *Methanosarcina barkeri* str. Fusaro (Feist et al., 2006). The RAVEN and COBRA Toolboxes (Agren et al., 2013; Heirendt et al., 2019) were used to generate the draft reconstruction. Each reaction in the draft model was evaluated for energy (ATP, NADH and NADPH accumulation) and mass balances as part of the quality control tests to guarantee model functionality and accuracy. Reactions associated with template genes were conserved in the first draft model to ensure model connectivity as well as the model’s capability to perform simulations. Nitrogen fixation and hydrogen consumption reactions were imported from the M-model templates. The resulting draft model contained 2,432 metabolic reactions and 1,918 metabolites divided into three different compartments (cytoplasm, periplasm, and extracellular space).

**Fig. 2:**
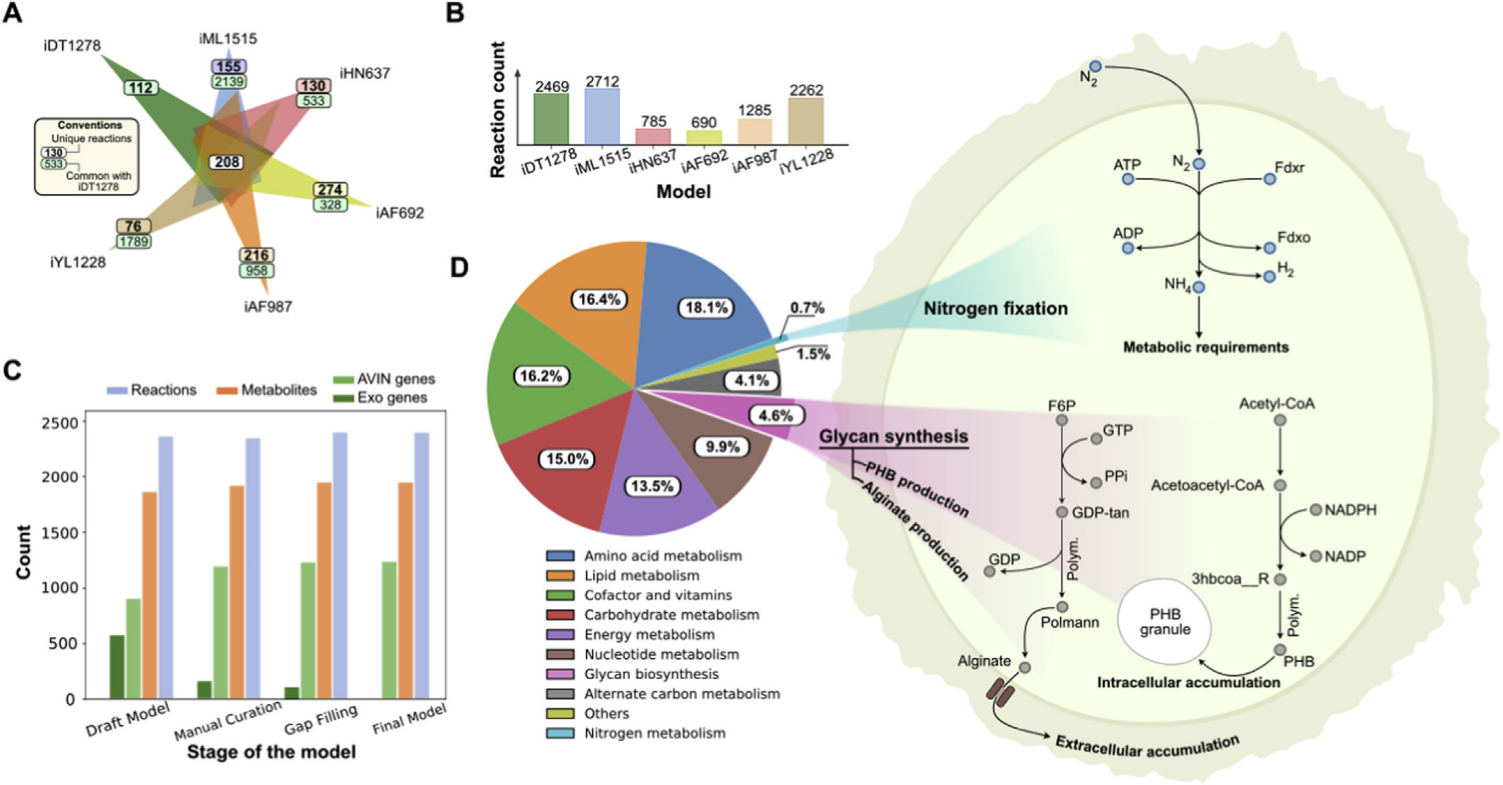
Characteristics of iDT1278. A) Comparison among the template models (*Escherichia coli* str. K-12 substr. MG1655, *Klebsiella pneumoniae* subsp. pneumoniae MGH 78578, *Geobacter metallireducens* GS-15, *Clostridium ljungdahlii* DSM 13528 and *Methanosarcina barkeri* str. Fusaro) and *i*DT1278 reactions. The six models share 208 core metabolic reactions. *A. vinelandii* DJ model contains 112 unique reactions related to aromatic compounds metabolism, alginate and PHB production, etc. B) Number of reactions in the template models and *A. vinelandii* DJ. C) Change in the number of reactions, metabolites and genes at the different stages of the reconstruction process/manual curation of *A. vinelandii* DJ. D) Reactions distribution through the subsystems in the genome-scale model; subsystems were grouped into 11 groups summarizing the complete metabolism of *A. vinelandii* DJ. Nitrogen fixation, PHB, and alginate production are highlighted in the condensed pathway diagrams. Alginate accumulation occurs in the extracellular space meanwhile PHB storage happens in the cytoplasm compartment. Nitrogen fixation in *A. vinelandii* can be performed by different specialized nitrogenases.

#### 3.1.1 Model refinement

Model refinement was performed using two principal steps: manual curation and gap filling. Every gene-protein-reaction (GPR) association was verified using multiple databases (e.g. KEGG, Biocyc, BRENDA, and MetaNetX) and available information from the literature. Manual curation was based on protein sequence similarity. The genes annotated in the GPR associations were aligned to protein sequences of *A. vinelandii*. Sequences, which passed the BLASTp parameters (see Methods), were assigned functionality based on information in the bioinformatics databases. The assigned genes in the GPR rules were replaced with the *A. vinelandii* genes (AVIN).

The original draft model consisted of 1,532 genes (Fig. 2A) corresponding to 934 AVIN genes and 598 genes from the template. At the end of the second manual curation, the AVIN genes increased to 1,233 and the total number of template genes was 102. Intuitively, as the level of curation increases the number of genes from template models decreases. When the genes in the model were curated by functionality, the number of genes from template models was zero.

#### 3.1.2 Gap filling

After the manual curation, the total number of reactions and metabolites in the model was 2,416 and 1,976, respectively. We used literature information and experimental data from the Biolog plates results (PM1 and PM2 for carbon sources and PM3 for nitrogen sources) to add or remove reactions in the model. Each reaction added to the model in this step was manually reviewed to maintain concordance in the GPR associations. Overall, a total of 51 reactions and 29 metabolites (mostly reactions related to carbohydrate and amino acids catabolism) were added to the model during the gap filling process (Fig. 1, Fig. 2C). The reactions added to the model were mainly transport and interconversion reactions to connect the carbon or nitrogen sources with intracellular metabolites from the model.

#### 3.1.3 Model properties

*The Azotobacter vinelandii* DJ metabolic model (*i*DT1278) consists of 2,003 metabolites, 2,469 reactions and 1,278 genes (around 26% of all annotated coding genes in the genome). Specific details about the reactions and metabolites from the model are summarized in Table S1 *i*DT1278 was validated using experimental data under nitrogen fixation (diazotroph) and ammonium assimilation (non-diazotroph) conditions. *i*DT1278 contains all the reactions and genes involved in nitrogen fixation, PHB, and alginate biosynthesis (Fig. 2B).

The properties of *i*DT1278 are shown in Fig. 2. Most of the reactions in the model belong to amino acid metabolism, lipid metabolism, and cofactor and vitamins metabolism (60% of total reactions of the model). Specific metabolic capabilities of *A. vinelandii* DJ such as nitrogen fixation (nitrogen metabolism), PHB and alginate production (glycan and secondary metabolites biosynthesis) represent around 3% of the metabolic reactions. Template models used during the reconstruction share 208 reactions with *i*DT1278. Most of the reactions (a total of 2,139 reactions) were taken from the first template (*Escherichia coli* K12 substr. MG1555, *i*ML1515) (Fig. 2A). Nitrogen fixation and H_2_ consumption pathways were obtained from the templates *i*HN637(*Clostridium ljungdahlii* DSM 13528) and *i*AF987 (*Geobacter metallireducens* GS-15). *i*DT1278 shares 208 reactions among all the template models (Fig. 2C) which are related to core metabolic pathways (TCA cycle, oxidative phosphorylation, amino acids metabolism, etc.). Table 1 shows a comparison of the properties of the different metabolic models reconstructed for *A. vinelandii*. As a result, *i*DT1278 represents, to our knowledge, the most comprehensive M-model of the diazotroph *A. vinelandii* available to date. However, the *A. vinelandii* metabolic model from CarveMe (Machado et al., 2018) contains the closest number of reactions, metabolites and genes using the BiGG database information.

**Table 1:** Comparison of the principal model properties (reactions, metabolites and genes) available for *A. vinelandii*.

Model provenience	Reactions	Metabolites	Genes	Reference
Present work	2,469	2,003	1,278	Present work
Model SEED	1,570	1,416	903	Henry et al. (2010)
CarveMe	2,422	1,978	1,395	Machado et al. (2018)
García (Alginate and PHB production)	46	39	0	García et al. (2018)
Inomura (Nitrogen fixation)	33	17	0	Inomura et al. (2018)

#### 3.1.4 Biomass objective function

The biomass objective function (BOF) contains the principal constituents and the abundance of each metabolite involved in biomass production. The proportion of each metabolite participating in the BOF composition is determined per gram of biomass. *i*DT1278 includes two biomass reactions: 1) An initial BOF was obtained from the first template (*Escherichia coli* K12 substr. MG1555, *i*ML1515) based on their physiological similarity (Gram-negative bacteria); the stoichiometric coefficients of the amino acids present in the BOF were calculated based on the theoretical amino acid abundance in the genome, using 55% of the biomass composition from amino acids. 2) A second BOF was determined from the first reaction to predict the alginate production since *A. vinelandii* DJ produces alginate only under specific metabolic conditions (Noar et al., 2015). The second BOF contains the same constituents present in the first BOF plus periplasmic alginate in order to simulate the complete metabolism and alginate production of *A. vinelandii*.

### 3.2 *i*DT1278 predicts accurately phenotypic experimental data

#### 3.2.1 Growth rates validation in carbon and nitrogen sources

**Fig. 3:**
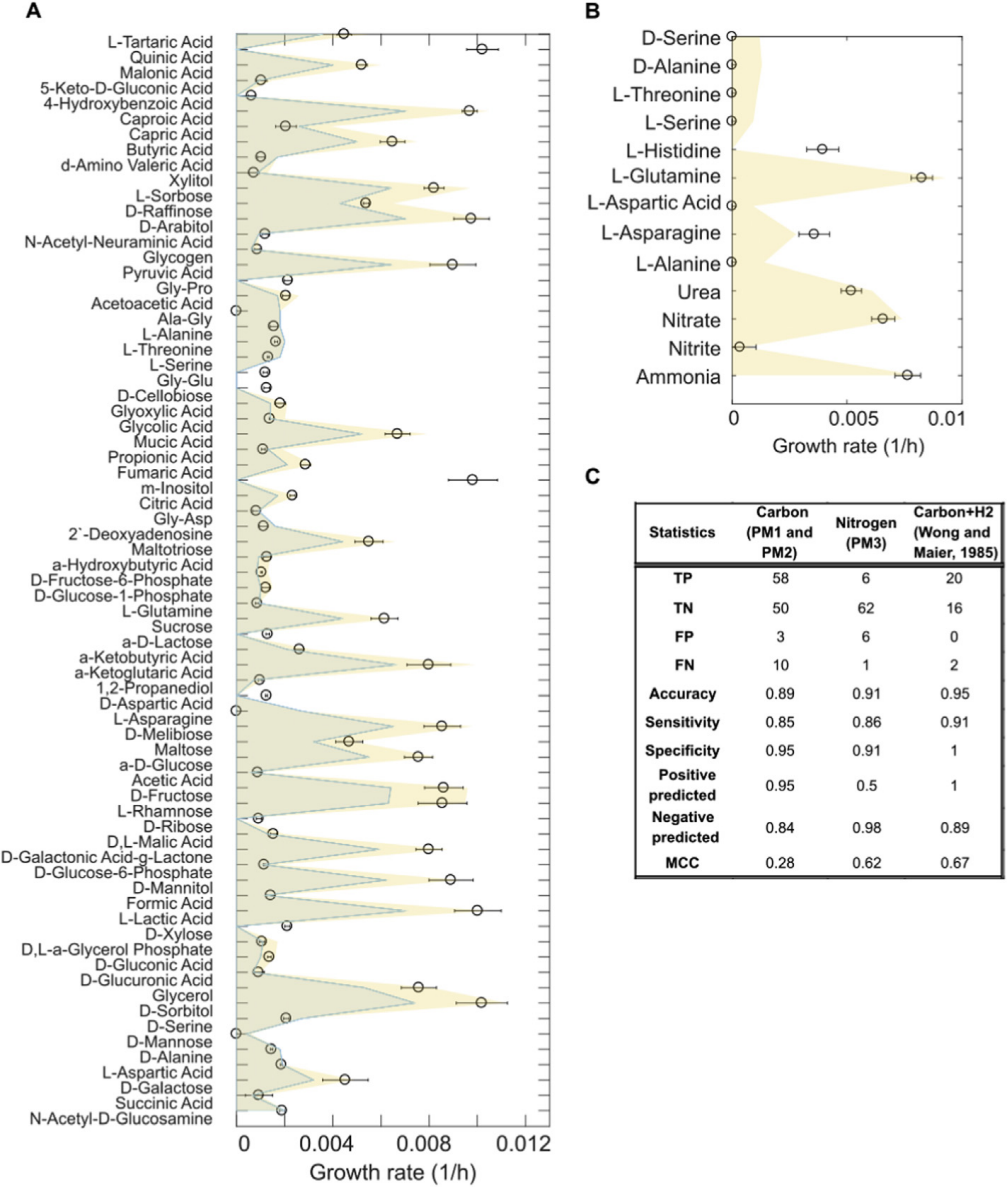
Model validation using high-throughput phenotypic data for different carbon and nitrogen sources. A) Predicted growth values of A. vinelandii DJ in nitrogen fixation (N_2_ ​+ ​Carbon source) or ammonium assimilation (NH_4_ ​+ ​Carbon source) conditions using 121 carbon sources (PM1 and PM2 from Biolog Plates). However, only 71 carbon sources are shown in Fig. 3. B) Estimated growth for 75 nitrogen sources, nevertheless just 12 nitrogen compounds are presented in the graph (PM3 from Biolog Plates). Pyruvate was used to constrain the model as the unique carbon source during the nitrogen validation. C) statistical parameters and Matthews correlation coefficient of the predictions for the carbon (first column) and nitrogen sources (second column) using the Biolog Plates information; true positive (TP), true negative (TN), false positive (FP) and false negative (FN). Statistical analysis of the estimations for 38 carbon sources under nitrogen fixation and H_2_ consumption conditions (third column). The data used during the simulations were obtained from Wong and Maier (1985).

The model was validated under a wide range of different growth conditions (diazotrophic and non-diazotrophic growth), using high-throughput phenotypic data as well as literature information. Initially, *i*DT1278 was tested under six different experimental conditions, specifically, carbohydrates under diazotrophic and non-diazotrophic conditions (Table 2). The M-model predicted precisely the growth rates for all the carbon sources using ammonium or molecular nitrogen as nitrogen sources. For the carbon sources in non-diazotrophic conditions (sucrose, mannitol and glucose), the predicted growth rates are consistent with experimental values obtained from the literature, resulting in an average accuracy close to 95%. For example, the predicted growth using mannitol (uptake rate of 0.83 mmol/gDW/h) as sole carbon source was 0.0472 1/h, agreeing with the experimental data (0.045 ​± ​0.003 1/h). Average precision under nitrogen fixation conditions decreased significantly to 83%. Table 2 shows the comparison between experimental data from literature and predicted values for *A. vinelandii* DJ. Initial results showed higher model accuracy (12% more) when predicting growth rates using ammonium as nitrogen source compared to N_2_. Subsequently, flux balance analysis (FBA, Orth et al., 2010) was performed for a group of 38 carbon sources in diazotrophic and H_2_-consuming conditions (Wong and Maier, 1985). Statistical results show for the subset of 38 carbon sources (Fig. 3C) an accuracy of 95%, with 20 true positive predictions (100% positive predicted) and 16 true negative predicted results (89% negative predicted). Matthews correlation coefficient (MCC) was calculated under the conditions previously mentioned, obtaining a value of 0.67 (Fig. 3C). The false negative predictions obtained during the validation of aconitate and lactose are related to the absence of literature information about the enzymes which metabolize the carbon sources into familiar metabolites for the microorganism (e.g. the model lacks enzymes to convert lactose into glucose and fructose).

**Table 2:** Predicted and experimental growth rates reported for *A. vinelandii* DJ under different carbon and nitrogen sources.

Carbon source	Nitrogen source	Experimental value (1/h)	Predicted growth (1/h)	Reference
Glucose	Ammonium	0.0505	0.0486	Clementi (1997)
Mannitol	Ammonium	0.045 ​± ​0.003	0.0472	Revin et al. (2018)
Sucrose	Ammonium	0.076 ​± ​0.004	0.07	Díaz-Barrera et al. (2016)
Glucose	Nitrogen	0.06 ​± ​0.0002	0.09	Wong (1988)
Fructose	Nitrogen	0.048 ​± ​0.002	0.0517	Wong (1988)
Galactose	Nitrogen	0.074 ​± ​0.007	0.065	Wong (1988)

Additional experimental validation was performed using Biolog plates for a set of carbon (PM1 and PM2) and nitrogen (PM3) sources to determine the growth rate values of *A. vinelandii* DJ. Out of 190 carbon sources from the Biolog plates, 123 compounds were identified in the model; the simulations were performed under two specific conditions: diazotrophic and non-diazotrophic simulation conditions. However, experimental results were obtained only under growth with ammonium as the unique nitrogen source. The same procedure used in PM1 and PM2 experiments was followed to estimate the growth rates with 75 different nitrogen sources. For this case, simulations were performed using pyruvate as the carbon source. Fig. 3 shows the complete analysis of the experimental and predicted data for all carbon (Fig. 3A) and nitrogen sources (Fig. 3B); statistical parameters (accuracy, sensitivity, specificity, positive predicted, negative predicted and Matthews correlation coefficient) were calculated for non-diazotrophic conditions. Subsequently, the same experimental results from the Biolog plates were used to calculate the statistical parameters of the CarveMe model to establish a comparison between *i*DT1278 and the CarveMe metabolic model accuracy. Details of CarveMe predictions and statistical parameters using Biolog plates data are presented in Table S3.

For carbon sources validation, an accuracy of 89% was achieved with 58 true positive predictions (95% positive predicted) and 50 true negative estimations (84% negative predicted). For this subset of compounds, accuracy decreased significantly in negative predictions (10 false negative estimations). These negative prediction disagreements involve carbohydrates and some amino acids as carbon sources. Some false negatives appear to be related to the lack of evidence about required transporters. Simulated growth rate values in ammonium assimilation conditions were significantly higher than in the nitrogen fixation conditions (>26%). Higher accuracy was observed in both positive and negative predictions in *i*DT1278 compared to CarveMe simulations (global accuracy of 61%, 72% positive predicted and 54% negative predicted). These statistical results support the clear differences between the quality predictions of *i*DT1278 and other metabolic models available for *A. vinelandii*. Significant changes in the growth rate values were not observed when using amino acids as carbon sources since these organic molecules contain nitrogen, which provides an indirect supply of this element to the microbe. For the nitrogen source validation, an overall accuracy of 91% was accomplished, nonetheless, the number of non-growing conditions increased considerably in comparison with the carbon condition experiments (almost 91% of nitrogen experiments resulted in no growth), indicating a reduced capability to grow in a wide range of nitrogen sources.

### 3.3 Carbon and nitrogen partitioning analysis

The average metabolic fluxes of carbon and nitrogen elements were determined for all carbon sources with growth rate values greater than 0.001 from Biolog plates data (61 total). The complete dataset with the experimental data is summarized in Table S2. The metabolic fluxes for both elements were grouped in 43 specific subsystems to identify the activity of the main pathways for all the experimental conditions. The highest average carbon fluxes (see Methods) were obtained from energy metabolism (82 mmol/gDW/h), oxidative phosphorylation (67.5 mmol/gDW/h), biomass and maintenance functions (36 mmol/gDW/h), and TCA cycle (25 mmol/gDW/h) for both nitrogen (N_2_ and NH_4_) conditions (disregarding transport fluxes). Similar tendencies were observed for the internal nitrogen flux distributions across the subsystems for diazotrophic and non-diazotrophic growth. However, the average flux distributions of carbon and nitrogen decline under diazotrophic conditions. Regarding global metabolic fluxes, the global carbon flux drops 4.9% and the nitrogen global flux value decreases 5.5%. The pathways with higher variation between carbon and nitrogen fluxes in diazotrophic and non-diazotrophic conditions were riboflavin metabolism (45%), glycine, serine and threonine metabolism (22%), alanine, aspartate and glutamate metabolism (26%), and nucleotide synthesis (10%). These subsystems are well-known for containing metabolites with high nitrogen content. The decline in the carbon and nitrogen global flux values of these pathways can be related to the low available nitrogen under diazotroph conditions due to the high energy cost of nitrogen fixation.

**Fig. 4:**
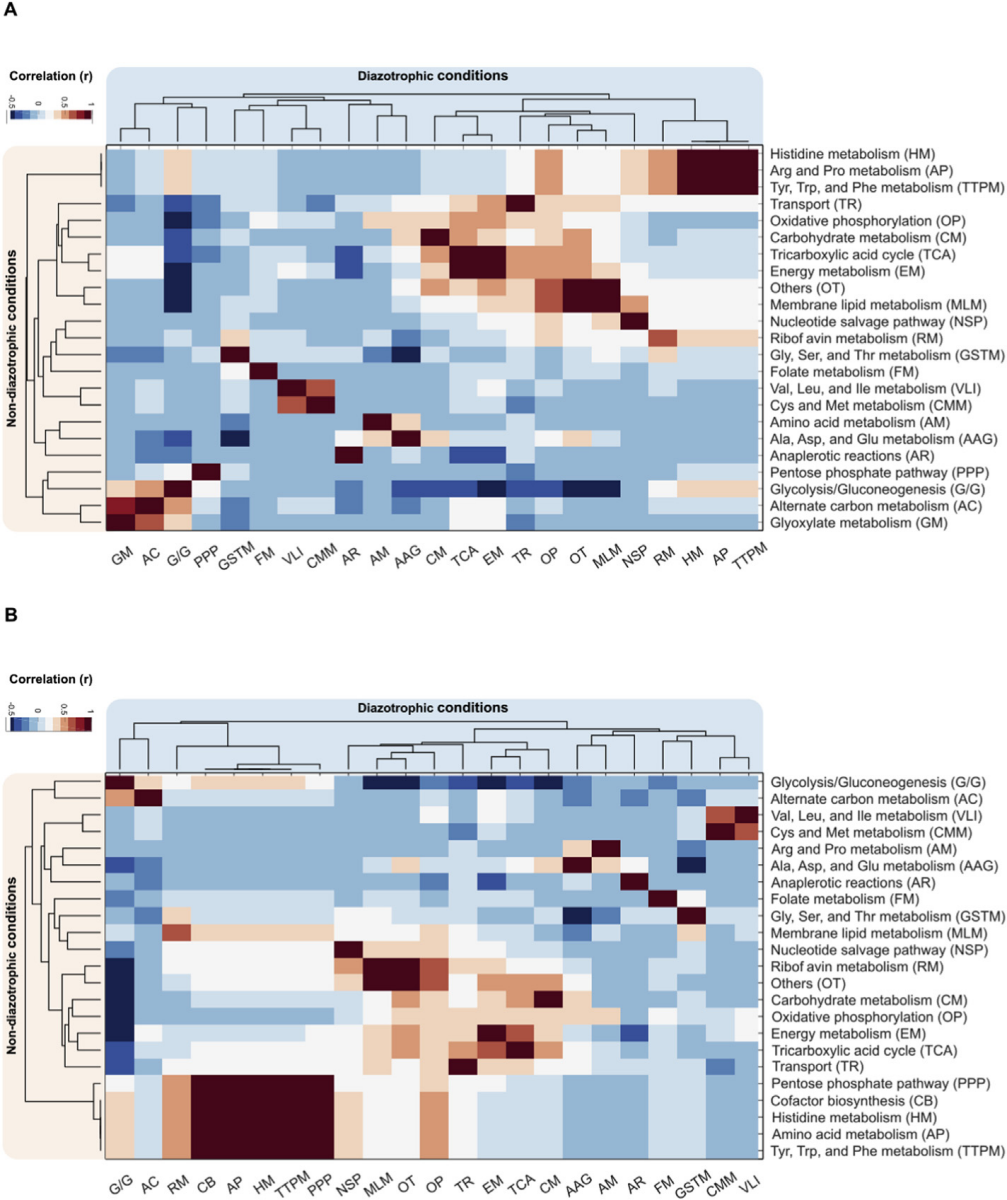
Carbon and nitrogen partitioning distribution from Biolog plates experimental data. A) Average carbon flux correlation coefficients through all subsystems in diazotrophic and non-diazotrophic conditions; B) average nitrogen flux correlation coefficients through all subsystems in diazotrophic and non-diazotrophic conditions.

The grouped average fluxes of carbon and nitrogen elements per subsystem calculated for diazotrophic and non-diazotrophic conditions were compared through a linear correlation analysis to determine how the subsystem flux values behave under both nitrogen (N_2_ and NH_4_) conditions. Fig. 4 presents the linear correlations values between diazotrophic and non-diazotrophic conditions of the active subsystems. For carbon and nitrogen partitioning analysis, the highest correlation coefficients (Pearson correlation >0.95, p-value <1 ​× ​10^−30^) were observed in all the amino acids pathways, biomass and maintenance functions, energy metabolism, carbohydrate metabolism, and some subsystems related to lipid metabolism (lipopolysaccharide biosynthesis and glycerophospholipid metabolism), demonstrating similar metabolic flux distributions through these specific subsystems when comparing diazotrophic and non-diazotrophic conditions. However, weak correlation values were obtained in riboflavin metabolism (*r* ​= ​0.66), oxidative phosphorylation (*r* ​= ​0.32 in carbon fluxes and *r* ​= ​0.37 in nitrogen distributions) and transport to the inner membrane (*r* ​= ​0.12 and *r* ​= ​0.31, respectively), showing an average flux distribution decrease of 45% for these specific subsystems when comparing carbon and nitrogen activity in diazotrophic against non-diazotrophic conditions. Additionally, strong correlation values were observed in specific subsystem clusters for both nitrogen conditions.

### 3.4 Alginate and PHB production estimated through *in-silico* experiments

*A. vinelandii* DJ contains specific mechanisms to produce and secrete alginate into the extracellular space. In *i*DT1278 we manually curated six specific reactions related to alginate biosynthesis including 16 different genes. One of the most important reactions involved in this pathway is the alginate epimerase (EC 5.1.3.37), which encompass a complex protein system to synthetize the alginate polymer (Pacheco-Leyva et al., 2016).

We evaluated the model accuracy to growth and alginate production using four carbon sources under diazotrophic and non-diazotrophic conditions. Experimental data retrieved from the literature (Revin et al., 2018) was only available for growth with ammonium. Simulations were confirmed to accurately predict (true positive predictions) alginate production rates with three carbon sources (glucose, mannitol, and sucrose). For example, the predicted growth using glucose (uptake rate of 0.33 mmol/gDW/h) as the sole carbon source was 0.1478 1/h and an alginate production rate of 0.25 mmol/gDW/h, agreeing with the experimental data (growth rate of 0.152 ​± ​0.012 1/h and alginate production rate of 0.265 ​± ​0.018 mmol/gDW/h). Lactose was the only compound with a mismatch between reported values and *in-silico* outputs (false negative). The disagreement appears to be associated to the lack of information in the literature and bioinformatic databases about the essential enzymes in the metabolism of this disaccharide in *A. vinelandii* DJ.

We compared the growth rates determined under diazotrophic and non-diazotrophic conditions for alginate production. The simulation analysis showed a significant increase in the growth rates (close to 28%) when the *A. vinelandii* consumes ammonium as a nitrogen source instead of molecular nitrogen. Additionally, a second comparison of alginate production rates between ammonium and molecular nitrogen exhibited the same increasing tendency when ammonium is used as a unique nitrogen source instead of molecular nitrogen (around 27%).

**Fig. 5:**
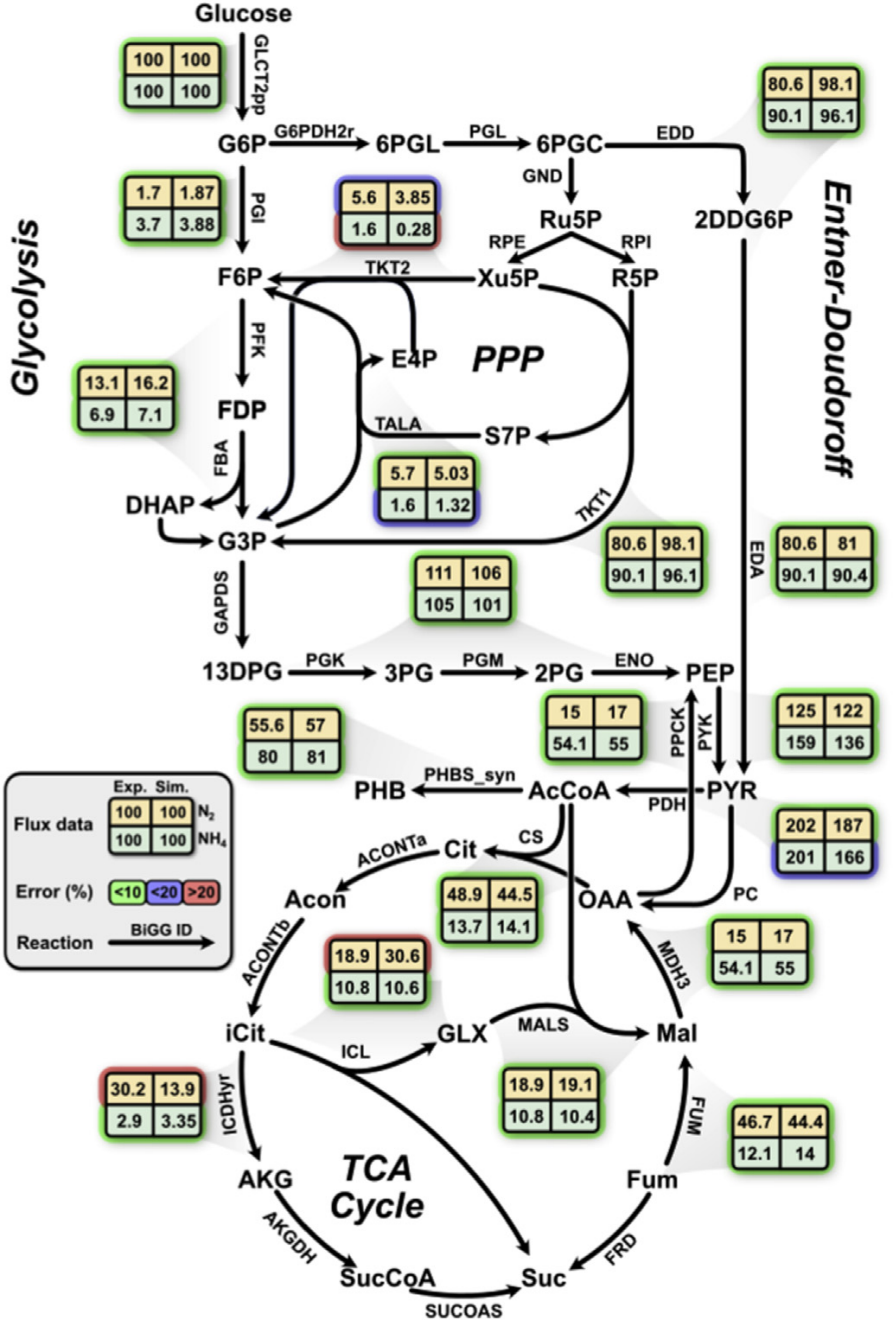
Metabolic flux map distribution of A. vinelandii under diazotrophic and non-diazotrophic conditions. The map displays major metabolic pathways involved in the PHB production. Values of metabolic flux are normalized to a glucose uptake rate of 100. Predicted metabolic fluxes were compared against fluxomic data determined by Wu et al., in 2019. The reactions were labeled according to their percent error (green, blue and red) and nitrogen source (green and yellow). Abbreviations: G6P, glucose-6-phosphate; F6P, fructose-6-phosphate; FDP, fructose-1,6-bisphosphate; G3P, 3-phosphoglycerate; DHAP, dihydroxyacetone phosphate; 13DPG, 3-phosphoglyceroil phosphate; 2 ​PG, 2-phosphoglycerate; PEP, phosphoenolpyruvate; PYR, pyruvate; Cit, citrate; Acon, aconitate; iCit, isocitrate; AKG, a-ketoglutarate; sucCoA, succinyl coenzyme A; Suc, succinate; Fum, fumarate; Mal, malate; OAA, oxaloacetate; GLX, glyoxylate; PHB, polyhydroxybutyrate; 6PGL, 6-phospho-glucono-1,5-lactone; 6PGC, 6-phospho-gluconate; 2DDG6P, 2-Dehydro-3-deoxygluconate 6-phosphate; Ru5P, ribulose-5-phosphate; R5P, ribose-5-phosphate; S7P, sedoheptulose-7-phosphate; X5P, xylulose-5-phosphate; E4P, erythrose-4-phosphate. (For interpretation of the references to colour in this figure legend, the reader is referred to the Web version of this article.)

While alginate is transported to the extracellular space, PHB is intracellularly stored (Yoneyama et al., 2015). To simulate PHB accumulation in *A. vinelandii* we incorporated a sink reaction to the model *i*DT1278. Simulated flux distributions about PHB production were validated using fluxomic data retrieved from Wu et al. (2019). The metabolic fluxes of the reactions involved in the PHB synthesis and related pathways (glycolysis, pentose phosphate pathway, the Entner-Deundoroff pathway, and the TCA cycle) were calculated through FBA for diazotrophic and non-diazotrophic conditions. The simulation results were compared with the experimental measured fluxes (Wu et al., 2019) and the percent error was estimated by reaction (Fig. 5). A general agreement in the reaction fluxes was observed under both nitrogen (N_2_ and NH_4_) conditions. A total of 16 out of 19 reaction flux estimations presented a global accuracy above 90% for diazotrophic and non-diazotrophic conditions. Disagreements were detected in three specific reactions: the transketolase (TKT2), aconitase (ACONTa/ACONTb) and isocitrate dehydrogenase (ICDHyr) in which the percent errors were above 20%. According to the predicted and experimental results, a higher PHB production is obtained under non-diazotroph conditions due to higher energy cost for nitrogen fixation. Additionally, most of the carbon coming from the glucose is metabolized through the Entner-Doudoroff (ED) pathway, generating less energy than glycolysis, but producing pyruvate (PHB precursor) in fewer steps. The available pyruvate generated though the ED pathway is mostly used in the TCA cycle and the PHB synthesis (Castillo et al., 2013; Wu et al., 2019), allowing the growth of the microorganism and the production of this biopolymer.

## 4 Discussion

### 4.1 Model reconstruction

Here we have created the first comprehensive genome-scale metabolic model for *A. vinelandii* DJ (*i*DT1278) that focuses on nitrogen assimilation, nitrogen fixation, as well as on alginate and PHB production. The model consists of 1,278 genes involved in 2,469 reactions. Compared to the first microorganism template used in the present work (*Escherichia coli* str. K-12 substr. MG1655, *i*ML1515), the percentage of metabolic genes per genome decreases from 31% (*i*ML1515) to 26% (*i*DT1278). However, a higher percentage of metabolic genes (from 26% of *A. vinelandii* to 12% of the average genes in the photosynthetic models) was observed when *i*DT1278 was compared to three photosynthetic organisms in the BiGG database: *C. vulgaris* UTEX 395, *i*CZ843 (Zuñiga et al., 2016), *Synechocystis* sp. PCC 6803, *i*JN678 (Nogales et al., 2012) and *C. reinhardtii*, *i*CR1080 (Chang et al., 2011). The M-model is accurate to 89% for all the carbon sources and 91% for nitrogen sources. The model was validated using a wide variety of carbon (159 compounds) and nitrogen (75 metabolites) sources. *i*DT1278 shown a significant higher accuracy (27% upper) in the predictions compared to the CarveMe model simulations. Additionally, *i*DT1278 predicted accurately the growth ratio and production values of alginate and PHB production under diazotrophic and non-diazotrophic conditions. To our knowledge, this is the first M-model at genome-scale capable to simulate several carbon and nitrogen conditions (close to 250 conditions) with a high precision even when comparing internal metabolic fluxes.

### 4.2 Model validation

#### 4.2.1 Nitrogen fixation

*i*DT1278 accurately predicts the growth of *A. vinelandii* using different carbon sources under diazotrophic and non-diazotrophic conditions. The model contains all required reactions and constraints to successfully simulate the BOF representing the growth of the organism. Model predictions have been confirmed by experimental validation using Biolog plates (PM1, PM2 and PM3). With this information we elucidated the preferred mechanism used by the *A. vinelandii* DJ to fix nitrogen while growing with different carbon sources. The N_2_ uptake depends on the ammonium concentration and metal cofactor concentrations required for the nitrogenases (García et al., 2018; Inomura et al., 2018). In our model, a difference in growth rate can be observed under diazotrophic and non-diazotrophic conditions. The growth rate under different carbon sources is higher during NH_4_ assimilation than during N_2_ fixation. However, the growth rates are quite similar for growth with amino acids under diazotrophic and non-diazotrophic conditions. This can be explained by the fact that amino acids release ammonium when metabolized which then becomes readily available to the organism. N_2_ fixation on the other hand is an ATP-dependent process and the organism must employ more energy to convert the N_2_ into ammonium.

Growth rate values decrease considerably when *A. vinelandii* DJ grows diazotrophically but nitrogen and carbon flux distributions per subsystem (e.g. amino acid, lipid, and carbohydrate metabolism) behave very similar in both nitrogen conditions. These flux distribution correlations suggest that the major discrepancies in the metabolism of *A. vinelandii* fixing nitrogen are related to the cofactor and vitamin pathways (riboflavin metabolism) and oxidative phosphorylation (energy generation). According to the model predictions (*i*DT1278), the vitamin, cofactor and oxidative phosphorylation pathways in *A. vinelandii* are involved in the generation of the precursors for the BOF; when ammonium is used as a nitrogen source, the carbon and nitrogen fluxes in these pathways are significantly higher (30%) than the fluxes estimated using molecular nitrogen as nitrogen source.

### 4.3 Alginate production

Alginate represents an important exopolysaccharide for *A. vinelandii* and is synthetized to reduce oxygen availability and thus increase nitrogenase activity for enhanced nitrogen fixation (Galindo et al., 2007; Nosrati et al., 2012). Additionally, this polymer has industrial relevance to multiple fields such as in pharmaceutical (Azevedo et al., 2014), biotechnological (Tomida et al., 2010), and food industry applications (Kuda et al., 1998). Therefore, elucidating the mechanism of alginate production could potentially provide insights for increasing production of this valuable biopolymer. *i*DT1278 accurately predicts three out of four carbon sources capable of producing alginate and the accompanying nitrogen sources to maximize biosynthesis of this polymer. The model also successfully predicts the decline in the growth and alginate production when molecular nitrogen is used as a nitrogen source. Alginate metabolism has been studied using genetic and regulation approaches to explain the synthesis of this valuable biopolymer, since most of the genes involved in this metabolic pathway are regulated by the presence of oxygen (Ertesvåg et al., 1995; Lloret et al., 1996; Núñez et al., 2000). In the present work we show that alginate production can also be explained based exclusively on metabolic requirements using mathematical and metabolic representations.

### 4.4 PHB production

PHB, like alginate, is synthesized by *A. vinelandii* to reduce oxygen availability and promote nitrogen fixation. *i*DT1278 contains all the genes and specific reactions involved in the production of PHB. The polymer is a high value product used in the production of biodegradable plastics and other environmental friendly polymers (Galindo et al., 2007). *i*DT1278 accurately predicts most of the metabolic flux values in the reactions (85% of the reactions) involved in PHB synthesis and pathways related to the generation of PHB precursors and energy metabolism (glycolysis, pentose phosphate pathway, the Entner-Doudoroff pathway, and the TCA cycle) for diazotrophic and non-diazotrophic conditions. The model is capable of accurately simulating all the variations presented in the flux distribution values for every reaction involved in the PHB synthesis under different conditions (ammonium or molecular nitrogen as unique nitrogen sources) using only metabolic data (stoichiometry and biochemistry data).

As with alginate production, PHB metabolism in *A. vinelandii* has been well studied using exclusively genetic and regulation perspectives since PHB accumulation mainly occurs during oxygen limitations (Castillo et al., 2013; Vijayendra et al., 2007); however, PHB metabolic distribution and biosynthesis can also be described using metabolic data according to the predictions performed by *i*DT1278. Our model could as a result be deployed to potentially optimize PHB production in *A. vinelandii.*

### 4.5 Network properties

Most of the False Negative (6 out of 10 total) and False Positive (6 total) predictions from the model related to carbohydrates and amino acids substrates could not be confirmed to belong to a pathway or to an enzyme that converts these carbon sources to internal metabolites present in the model either from literature information or available genomic and metabolomic databases. Moreover, when using amino acids as growth substrates, the model predicts very similar growth rate values for diazotrophic and non-diazotrophic conditions since some amino acids can release ammonium further supporting growth. These discrepancies between experimental evidence and model predictions could be related to the limited information available concerning specific metabolic mechanisms for metabolizing carbon sources as carbohydrates, lipids and amino acids in *A. vinelandii* or processes related to genetic, transcriptional and regulation. Indeed, while nitrogen metabolism is tightly regulated in *A. vinelandii* (Hamilton et al., 2011; Toukdarian and Kennedy, 1986), transcriptional and translational regulation is not currently part of this M-model but could in part be recapitulated by including all macromolecular synthesis as part of a subsequent ME-models (Liu et al., 2019, 2014; O’Brien et al., 2013). Furthermore, nitrogenase activity and activity of other nitrogen-associated pathways are also regulated at the enzyme level (Pacheco-Leyva et al., 2016), potentially also contributing further to the lack of agreement between model predictions and experimental data.

### 4.6 Future aspects

Insight into the metabolic processes using the genome-scale model *i*DT1278 could benefit low-cost media optimization for *A. vinelandii* and to further improve production processes as PHB, alginate and other biopolymers with high industrial value. Although this environmentally important model organism has been isolated and studied for more than 100 years, its physiology and some metabolic pathways have yet to be fully understood. We believe the current model provides a valuable step along the path towards better characterization of this important microbe in isolation as part of a microbial community (Tan et al., 2015; Zuñiga et al., 2019). Unraveling this knowledge for *A. vinelandii* could improve its use in industrial applications (Noar and Bruno-Bárcena, 2018), and the systems biology approaches presented here may provide a tool to help in achieving that goal.

## Author contributions

CZ, AZ, and KZ conceived the study. DT and CZ reconstructed the model and performed all simulations. DT and AP performed manual curation. JDT performed growth experiments. CZ, DT, AP, JDT, and MJB discussed the results. DT, AP, CZ, and KZ wrote the manuscript with input from all co-authors.

## Funding

This material is based upon work supported by the 10.13039/100000001National Science Foundation under Grant No. 1332344 and CBET-1804187, and the 10.13039/100000015U.S. Department of Energy (10.13039/100000015DOE), 10.13039/100006132Office of Science, Office of Biological & Environmental Research under Awards DE-SC0012658 and DE-SC0019388. D.T. was in part supported by Mexican 10.13039/100013101National Research Council, 10.13039/501100003141CONACYT, fellowship No. 932962.

## CRediT authorship contribution statement

**Diego Tec Campos:** Investigation, Methodology, Writing - original draft. **Cristal Zuñiga:** Conceptualization, Supervision, Project administration. **Anurag Passi:** Data curation, Writing - review & editing. **John Del Toro:** Investigation. **Juan D. Tibocha-Bonilla:** Validation. **Alejandro Zepeda:** Validation, Funding acquisition. **Michael J. Betenbaugh:** Investigation, Funding acquisition. **Karsten Zengler:** Conceptualization, Funding acquisition.

## Declaration of competing interest

The authors declare that they have no known competing financial interests or personal relationships that could have appeared to influence the work reported in this paper.

## Research data for this article

iDT1278 is available in. mat and. xml format as well as the carbon and nitrogen partitioning analysis in the repository from Zengler Lab: https://github.com/cristalzucsd/AvinelandiiDJ.
